# Electrospun Fibers of Cyclodextrins and Poly(cyclodextrins)

**DOI:** 10.3390/molecules22020230

**Published:** 2017-02-03

**Authors:** Alejandro Costoya, Angel Concheiro, Carmen Alvarez-Lorenzo

**Affiliations:** Departamento de Farmacología, Farmacia y Tecnología Farmacéutica, R+D Pharma Group (GI-1645), Facultad de Farmacia and Health Research Institute of Santiago de Compostela (IDIS), Universidade de Santiago de Compostela, 15872 Santiago de Compostela, Spain; ale.costoya@gmail.com (A.C.); angel.concheiro@usc.es (A.C.)

**Keywords:** electrospinning, cyclodextrin, polycyclodextrin, nanofiber, polypseudorotaxane, controlled release, regenerative medicine, filtration membrane, stimulus-responsive fiber, post-processing, fast dissolving amorphous products

## Abstract

Cyclodextrins (CDs) can endow electrospun fibers with outstanding performance characteristics that rely on their ability to form inclusion complexes. The inclusion complexes can be blended with electrospinnable polymers or used themselves as main components of electrospun nanofibers. In general, the presence of CDs promotes drug release in aqueous media, but they may also play other roles such as protection of the drug against adverse agents during and after electrospinning, and retention of volatile fragrances or therapeutic agents to be slowly released to the environment. Moreover, fibers prepared with empty CDs appear particularly suitable for affinity separation. The interest for CD-containing nanofibers is exponentially increasing as the scope of applications is widening. The aim of this review is to provide an overview of the state-of-the-art on CD-containing electrospun mats. The information has been classified into three main sections: (i) fibers of mixtures of CDs and polymers, including polypseudorotaxanes and post-functionalization; (ii) fibers of polymer-free CDs; and (iii) fibers of CD-based polymers (namely, polycyclodextrins). Processing conditions and applications are analyzed, including possibilities of development of stimuli-responsive fibers.

## 1. Introduction

Over the last decades, electrospinning has been recognized as a cost-effective and simple technique for producing continuous fiber structures from a wide variety of starting materials [1,2]. It relies on the application of a strong electric field to a pumped viscous polymer solution to induce its flight toward a collector whereas the solvent is being evaporated [3,4]. Importantly, the diameter of electrospun fibers, ranging from micro- to nanometers, provides mats of high surface area to volume ratio compared with other fiber production techniques. The capability of electrospinning to produce fibrous structures with high porosity and interconnected pores, as well as the malleability of obtained fibers conforming structures like sheets or tubes make electrospun fibers to find a broad spectrum of biomedical and non-biomedical applications [1,2,3,4,5,6,7]. The properties of electrospun fibers can easily be tailored to every particular purpose by tuning the composition (blending a variety of organic and inorganic materials in aqueous or organic solvents), electrospinning parameters, needle and collector configuration, and post-processing to endow them with further functionality [2,3,8,9]. Therefore, electrospun systems are useful in fields such as environmental engineering, defense and security, biotechnology, healthcare, biomedicine, drug delivery, filtration, nanocatalysis and nanoelectronics [7,10,11]. In the biomedical field, fibers of large-specific surface area with composition mimicking the extracellular matrix (ECM) serve as scaffolds for engineering of vascular, nerve, ligament and bone tissues and for wound healing [12,13,14,15,16]. Mats showing porous sheet-like structure can absorb wound exudates, protect from bacterial infections, maintain a moist environment and allow gas permeation [1,10,12]. Furthermore, the fibers can encapsulate large amounts of growth factors, enzymes and drugs useful for diverse therapies [1,4,14,16,17,18,19]. Alternatively, electrospun systems have been widely used as filtration membranes for biotechnology and environmental engineering applications. Fiber-based filters are effective size exclusion membranes for air and wastewater depending on their pore size, which should be adjusted to the fineness of particles to be filtered [20,21,22]. Electrospun membranes can be further functionalized by attachment of ligand molecules for affinity separation [21,23].

A wide range of materials have been successfully employed in electrospinning, from polymers to composites, semiconductors and ceramics [11]. Electrospinning requires compounds of certain high molecular weight with chains that can entangle when dispersed in the solvent (concentration above the critical overlapping value) and thus produce fibers when the electrical field-induced flow causes solvent evaporation; for this reason, polymers have been extensively electrospun [3,4,24,25,26]. The polymer nature and concentration influence viscosity, which is a critical solution parameter correlated with chain entanglement. According to polymer choice, the concentration should be regulated in order to obtain sufficient chain entanglement for fiber formation. Solution properties (viscoelasticity, conductivity, surface tension) are also determined by the solvent [3,4,27]. Variables of the process (applied electric field, tip-to-collector distance and flow rate of feeding) and the environment (temperature and humidity) have an impact on fibers formation and their morphology too [4,7,27].

The choice of the polymer is an important point for obtaining fibers that can meet the demands of each application. Usually, synthetic polymers exhibit reproducible behavior during electrospinning and provide highly uniform nanofiber mats. Hydrophobic synthetic polymers allow for the preparation of mats that maintain their structure upon contact with aqueous media, and thus they have been extensively tested for liquid filtration [3,7,8,23]. Biopolymers or naturally-occurring polymers have the advantages of being digestible or bioerodible and more biocompatible and less immunogenic than synthetic polymers. Thus, electrospun systems of proteins, polysaccharides, DNAs and lipids are attracting a great deal of attention in the food industry, cosmetic, biomedical and pharmaceutical fields [28]. Nevertheless, biopolymers have drawbacks such as poor mechanical properties and processing characteristics. Biopolymers act as thickening agents due to the ability of the chains to interact through weak but redundant multiple interactions that favor entanglement and even gel formation and, therefore, they confer high viscosity to solutions. Blends of different polymers have been extensively studied to overcome these limitations and to generate materials with desirable flow, surface tension and conductivity properties. Additionally, combinations of natural and synthetic polymers endow the fibers with the mechanical strength and cell affinity of the synthetic and natural polymers, respectively [3,8].

Although not polymers, cyclodextrins (CDs) exhibit appealing features when incorporated into electrospun fibers. These cyclic oligosaccharides form inclusion complexes with a variety of molecules, modifying the apparent solubility and stability of the guest molecule and, once incorporated into a polymer matrix, they can help tuning the release rate [29,30,31,32,33]. CDs and their inclusion complexes appear as an attractive tool for the design of fibrous materials with advanced performance. As an example, CDs forming inclusion complexes with volatile substances (e.g., essential oils such as geraniol, menthol or vanillin) can notably prolong the shelf-life of active food packaging based on electrospun mats [33]; whereas mats containing free CDs allow an efficient removal of hydrophobic organic molecules or heavy metals from air or solutions by forming of inclusion complexes [34]. Progress in this field is rapidly evolving with novel ways of incorporating CDs and exploiting their self-assembling properties into electrospun mats being explored.

The aim of this review is to provide an overview of the state-of-the-art on CD-containing electrospun mats. The information has been classified into three main sections: (i) fibers of mixtures of CDs and polymers, including polypseudorotaxanes and post-functionalization; (ii) fibers of polymer-free CDs; and (iii) fibers of CD-based polymers (namely, polycyclodextrins). Processing conditions and applications are analyzed, including possibilities of development of stimuli-responsive fibers.

## 2. Electrospun Mats of Mixtures of Cyclodextrins and Polymers

Electrospun systems containing CDs have been produced from native CDs or derivatives with a variety of polymers that provide electrospinnability to the blend and adequate properties to the mat for every specific application. It should be noted that in addition to the short size of CDs, the fact that some derivatives bear ionic groups (e.g., sulfobutylether-β-cyclodextrin, SBEβCD) hinders the formation of continuous fibers due to repulsive forces among the CDs; the presence of a shielding polymer being mandatory [35]. Conversely, non-ionic CDs have been shown able to improve the electrospinnability of ionizable polymers, such as chitosan [36].

### 2.1. Electrospining Methods for Cyclodextrin Incorporation

**Figure 1:**
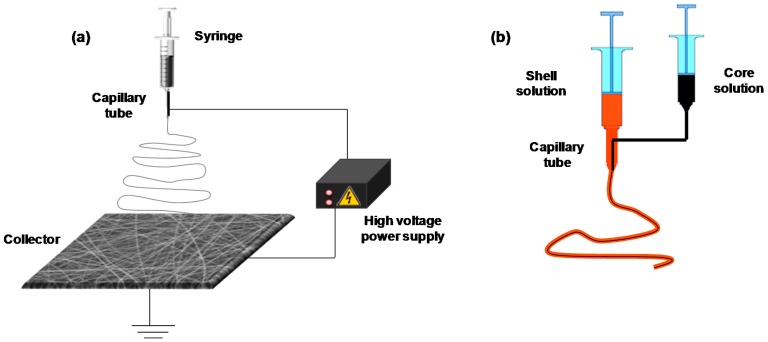
Electrospinning setup for the preparation of uniaxial fibers (**a**), and configuration of syringes and capillaries for preparation coaxial fibers (**b**).

The basic principle of electrospinning involves the application of a strong electric field to a metallic capillary tube (needle) where solution is pumped from a reservoir (syringe). The pendant droplet, held by surface tension forces, is electrified and deformed into a conical shape known as Taylor cone. When the applied electric force is increased above a threshold value needed to overcome the surface tension forces, a jet is ejected from the Taylor cone tip toward a grounded collector while the solvent is evaporated. Entanglements prevent breaking of jet that is subjected to stretching and bending instabilities. Thereby, solid fibers are deposited in the collector forming a non-woven mat [3,18,21]. A schematic setup of electrospinning is shown in Figure 1.

The electrospinning method may determine the location and accessibility to CD units in the obtained electrospun fibers, which has important consequences for potential applications. Most research aims to keep the CD cavities available to form inclusion complexes, although a few publications have dealt with the use of CDs as crosslinking agents in nanofibers [37]. As an example, electrospun fibers of an acrylic polymer and β-cyclodextrin (βCD) were post-processed under heating to induce dehydration of carboxylic groups of the polymer and esterification with hydroxyl groups of βCD. This post-spinning modification caused the fibers to become water-insoluble, which in turn facilitated their full development of reproducible pH-responsive swelling behavior [37,38].

#### 2.1.1. Uniaxial Processing

##### Blends of Cyclodextrins and Polymers

Within different electrospinning methods for mat manufacturing, the uniaxial electrospinning process is commonly used due to its relative simplicity. Electrospun mats are directly produced via electrospinning of solutions of pristine CDs or their derivatives solely or forming inclusion complexes with active agents, in the presence or absence of a polymer [5,39]. CD-containing fibers have been mostly electrospun with polymers that supply the required entanglements to facilitate uniform fiber formation [24,40]. Active ingredient (e.g., drug) distribution through electrospun fibers depends on its solubility in the processing solution. For example, incompatibility phenomena among the drug, the polymer and the solvent lead to non-homogeneous distribution of the active agent, which usually migrates to the fibers surface (flocculation effect), resulting in burst release when the fibers enter into contact with an aqueous medium [5,17,19,41]. Interestingly, inclusion complex formation facilitates homogeneous guest molecule distribution through electrospun fiber matrix. Remarkably, poorly water-soluble active agents in the form of inclusion complexes can remain soluble and stable in aqueous medium and thus allow for the use of water as solvent. Despite more homogeneous distribution, reports on mats prepared with inclusion complexes usually show faster release of guest molecules compared to mats in which the active ingredient is free. Hydrophilic CDs facilitate the wetting of the mats and the penetration of water into the fibers, favoring fibers erosion/dissolution, whereas the complex formation increases the concentration of diffusible species within the mats [19,42,43]. All these processes favor active ingredient release. For example, faster release of naproxen from poly(ε-caprolactone) (PCL) nanofibers was observed when the fibers contained inclusion complexes compared to those prepared with the free drug [42]. Polyvinyl alcohol (PVA) nanofibers loaded with curcumin-βCD inclusion complex have been reported to be more uniform and smooth than those with free curcumin, which was ascribed to the higher solubility of the inclusion complex in the preparation solvent [43]. Increasing the inclusion complex/PVA ratio, faster drug release was observed, which can be explained by the role that the concentration gradient plays on diffusion-driven release [44]. Conversely, release profiles from free curcumin/PVA fibers evidenced faster release from fibers prepared with low contents in curcumin, which facilitates drug solution in the polymer matrix and subsequent diffusion [4,19,43]. Similar results were reported for polylactic acid (PLA) nanofibers containing free gallic acid or gallic acid-hydroxypropyl-β-cyclodextrin (HPβCD) inclusion complexes when the release was evaluated in water or water/ethanol 90:10 *v/v* media [45]. Overall, inclusion complex enables the incorporation of higher contents in hydrophobic drug and accelerates drug diffusion from fibers once in aqueous medium [46]. For example, polyvinyl pyrrolidone (PVP) electrospun mats containing betel oil or clove oil forming complexes with HPβCD released 50% herbal oils in the first minute into contact with saliva and were shown useful for the treatment of *Candida*-associated denture stomatitis [47]. Fast dissolution fibers may also find applications as extemporaneous oral liquid formulations adapted to age requirements of pediatric and geriatric populations [48], or as sublingual formulations for treatment of acute disorders [49]. In addition to the improvement in dissolution rate, CDs are known to enhance drug permeation through biological membranes [50,51]. Simultaneous analysis of dissolution and permeation of aripiprazole from electrospun fibers of polyethylene glycol (PEG) and SBEβCD, using µFlux apparatus, revealed that the ultrafast complete dissolution of the drug from the fibers considerably enhances the flux through biological membranes compared to free drug and physical mixtures [35]. Moreover, permeation through the membrane attenuated the rate of drug precipitation in the supersaturated medium; drug molecules transferred to the receptor compartment lowered drug concentration in the dissolution (donor) compartment resembling the in vivo situation [35].

It should be noted that inclusion complex formation itself facilitates the electrospinning of highly volatile compounds, and also their retention into the fibers during storage or when exposed to the air [52]. Thereby, electrospun mats containing CDs have been investigated for encapsulation of flavors, fragrances and essentials oils that exhibit antibacterial, antioxidant or insecticidal properties, with potential application in active food packaging, cosmetics and medical devices [33,53,54,55]. Encapsulation in the CD cavities also provides protection against photodegradation [55], oxidation and thermal instability [56]. These advantages have prompted the use of electrospun mats also as skin nutrient-loaded facial masks [7,57]. Different formulations based on hydrophilic PVA and methylated βCD mats with ascorbic acid, retinoic acid, collagen and gold nanoparticles have been successfully prepared. Average fiber diameter was smaller in formulations including gold nanoparticles due to that the increase in charge density led to higher jet elongation [57]. Electrospun fibers can be wetted just immediately before application on the skin which is an advantage in terms of stability compared to pre-wetted conventional cotton face masks. Also the electrospun fibers fit better to the face topography and have a larger surface area in contact with skin, which favors skin permeation of the loaded compounds.

**Figure 2:**
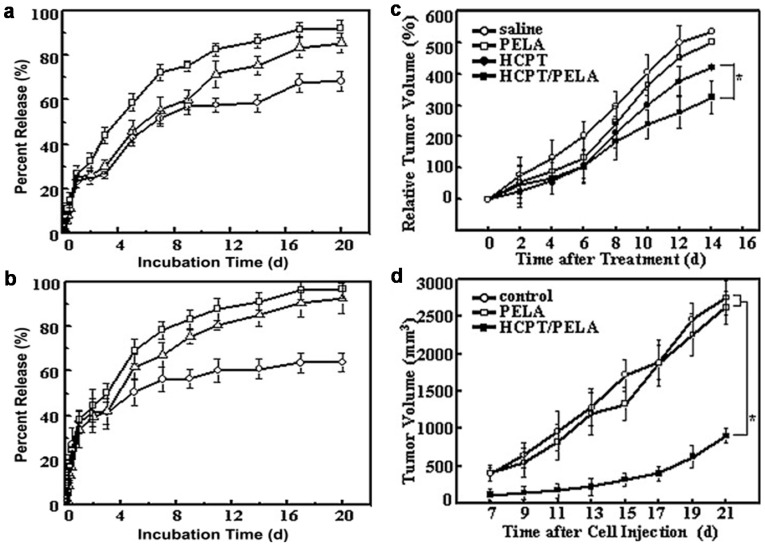
Release profiles of hydroxycamptothecin from poly(dl-lactic acid)-poly(ethylene glycol) (PELA) electrospun fibers containing (**a**) 1% or (**b**) 3% drug and various proportions in hydroxypropyl-β-cyclodextrin (HPβCD; 0.5% circles; 1.5% triangles; 2.5% squares), in phosphate buffer saline (PBS) medium at 37 °C. Tumor growth inhibition of H22 tumor bearing mice after intratumoral implantation of saline solution, free hydroxycamptothecin (HCPT) solution, blank PELA fibers and hydroxycamptothecin-loaded PELA fibers (**c**), and tumor development after injection of H22 tumor cells in a tissue pretreated with blank PELA fibers and hydroxycamptothecin-loaded PELA fibers or non-pretreated (control) (**d**). Reprinted from [61] with permission from Elsevier.

Several approaches have been tested to prolong drug release in aqueous medium [3,4,12]. Silk fibroin has been investigated to confer sustained release to electrospun fibers containing CD inclusion complexes of tamoxifen for local breast cancer therapy [58]. The fibers were made insoluble in water by spraying with ethanol or acetone, which decreased drug release rate. Electrospun fibers of hydroxypropyl cellulose (HPC) and HPβCD-sulfisoxazole complexes showing fast drug dissolution were sandwiched between two mats of PCL nanofibers (applying mild pressure for an efficient wrapping) [59]. The hydrophobic PCL barrier allowed more sustained sulfisoxazole release. Similarly, electrospun fibers of poly(ethylene oxide) (PEO), γCD and catalase were prepared in between PCL mats [60]. Compared to γCD/catalase physical mixture encapsulated in the PCL nanofibers, the sandwiched system showed higher stability but lower activity because of the slowed diffusion through the immobilizing fibers. Emulsion electrospinning has been shown suitable to provide sustained release of hydroxycamptothecin from fibers prepared from a water-in-oil emulsion of HPβCD-hydroxycamptothecin in poly(dl-lactic acid)-poly(ethylene glycol) (PELA) dispersion [61]. Importantly, the encapsulated drug retained unaltered the lactone ring required for the antitumoral activity. Intratumoral implantation studies evidenced that the drug-loaded fibers notably attenuated tumor growth once the tumor had already developed. Also, the fibers can exert protective effects against tumor development when implanted in a tissue before the tumor cells arrive [61] (Figure 2).

Uniaxial electrospun mats are also being investigated for sensing applications because the large surface area could improve the sensitivity of sensors and biosensors [1,6,21,62]. For example, multi-functional polyvinylidene fluoride (PVdF) membranes were developed with electroactive and electrocatalytic properties due to the presence of carbon nanotubes and Au particles [63]. Nanofibers were prepared from blends of PVdF with multiwalled carbon nanotubes (CNTs) and inclusion complexes between βCD and 4-aminothiophenol. βCD facilitated the dispersion of CNTs in the electrospinning blend, while 4-aminothiophenol favored gold nanoparticle nucleation onto the fiber surface during a post-spinning process. CNTs made the dispersions more conductive and thinner fibers were obtained. These electrospun mats were shown to be useful for the preparation of electrocatalytical electrodes. Also the incorporation of silver nanoparticles has received great attention because their catalytic and antiseptic properties make them applicable for sensing, wound healing and other biomedical uses [64,65,66]. Electrospun PVA/HPβCD nanofibers incorporating Ag nanoparticles were successfully electrospun [65]. PVA acted as reducing and stabilizing agent for obtaining of nanoparticles and further as polymeric matrix for electrospinning. HPβCD was also incorporated as additional reducing and stabilizing agent, which prevented nanoparticles aggregation and enhanced antimicrobial effects. Electrospun fibers containing high amounts of HPβCD notably inhibited the growth of *Escherichia coli* and *Staphylococcus aureus* [65]. Immunosensor chips have been fabricated by deposition onto gold-coated glass of poly(acrylic acid) (PAA)-βCD fibers that were heated for inducing the cross-linking of the polymer with βCD, which in turn improved the stability of the chip. Finally, the fibers were coated with multilayers of PAA and poly(diallyldimethylammonium chloride) (PDADMAC) and then decorated with monoclonal antibodies. Compared to chips without electrospun fibers, the prepared chips provided enhanced sensor signal [67].

Regarding separation applications, electrospun membranes require the functionalization with capturing agents (ligands) for the entrapment of specific molecules (affinity membranes). Functionalization with CDs allows for an efficient removal of hydrophobic organic molecules or metal ions from air or liquid solvents through inclusion complex formation [7,21,34,68,69,70,71]. Polymer component has to be carefully chosen for each specific application in order that the electrospun mat does not collapse or dissolve in contact with the filtered medium. For example, electrospun membranes of hydrophobic poly(methyl methacrylate) (PMMA) blended with βCD derivatives were developed to capture phenolphthalein from aqueous medium, preserving membrane integrity for long time [70]. For air filtration, affinity membranes were also produced using polymers of diverse nature or even polymer-free CD-containing mats [34,72]. Nylon 6,6 nanofibers incorporating βCD could efficiently capture toluene from vapors [72], while polystyrene mats containing α-, β- or γCD efficiently sorbed Cu(II) ions [73].

##### Polypseudorotaxanes

**Figure 3:**
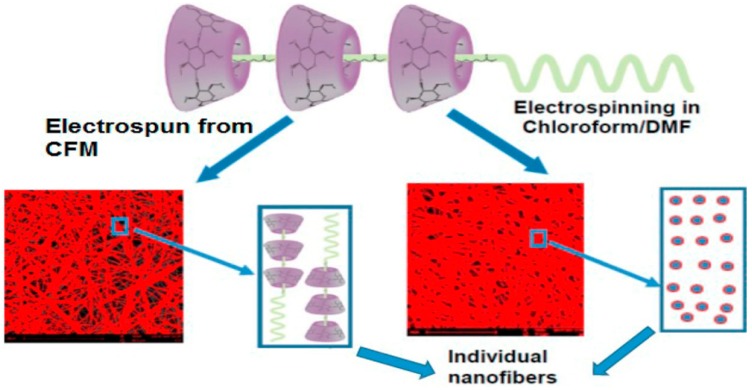
Electrospinning of α-cyclodextrin-poly(ε-caprolactone) (αCD-PCL) pseudorotaxanes dispersed in chloroform (CFM) led to fibers that retained the polypseudorotaxane structure, while in chloroform/dimethylformamide medium dethreading occurred and the fibers were formed by the individualized components. Reprinted with permission from [84]. Copyright (2016) American Chemical Society.

It should be kept in mind that when a polymer is mixed with CDs in a liquid solvent it is quite likely that the CD units thread along the main chain or side chains of the polymer forming polypseudorotaxanes [74,75]. The polypseudorotaxanes can assemble forming supramolecular structures in which crystalline-type interactions among threaded CDs play a relevant role; the arrangement of the polymer chains in the formed 3D network notably modify the properties of the system [76,77]. Polypseudorotaxanes can be formed accidentally or in purpose in the electrospinning solvent and they can remain intact in the fibers or be destroyed during electrospinning. Interactions between PEG and αCD were the first in being described [78] and widely applied for many purposes [51,79]. Nevertheless, the crystalline structure of αCD-PEG polypseudotoxane assemblies is so strong that electrospinning is not possible. To solve this problem these polypseudorotaxanes were diluted in PEO solutions at various weight ratios. Aqueous dispersions containing the same weight percentage in PEO and in polypseudorotaxanes (4% *w/v* each) led to nanofibers of 160 nm with few beads. Large beads were observed for higher polypseudorotaxane concentration because of deficient stretching of the assemblies during electrospinning [80]. αCD can also thread along PCL altering the crystallization of the polymer, which is not modified by γCD [77,81]. Interestingly the interaction can be tuned using mixtures of solvents that differ in solubilization capability for αCD and PCL. Free CDs in non-stoichiometric complexes improve the hydrophilicity of PCL nanofibers [82]. Similarly to the case of αCD-PEG polypseudotoxane, stoichiometric αCD-PCL polypseudotoxanes can be incorporated into electrospun mats after dilution in PCL solutions. For example, 10% polypseudorotaxanes in 14% PCL solution rendered bead-free fibers with remarkably higher tensile strength than PCL solely fibers [83]. It was shown that non-stoichiometric αCD-PCL polypseudotoxanes dispersed in chloroform retain their structure in the electrospun fibers, but when a chloroform/dimethylformamide mixture is used αCD abandons the polymer (Figure 3). The former fibers showed improved mechanical properties [84]. Similarly electrospinning of βCD and PCL mixtures in chloroform:dimethylformamide solvent led to nanocomposites having βCD cavities free and unthreaded by PCL and thus suitable for removing wound odors [85]. Direct electrospinning of αCD-PCL polypseudotoxanes was also possible using DMSO/CH_2_Cl_2_ (3/2, *v/v*); the obtained fibers exhibited the reactive hydroxyl groups of CDs at the surface available for further conjugation with active substances. These electrospun fibers performed quite well as scaffolds for cell growth and osteogenic differentiation in vitro, showing enhanced production of collagen [86].

##### Post-spinning Modifications

Drug release kinetics from electrospun fibers is predominantly regulated by diffusion although it can also be controlled via degradation/erosion of the matrix [44,87]. Post-spinning modifications such as chemical or plasma treatments may help to prolong the drug release process [5,88]. In that way, diffusivity could depend on the concentration gradient across the fibers or an external barrier or on the degradation/erosion of a coating layer [44].

**Figure 4:**
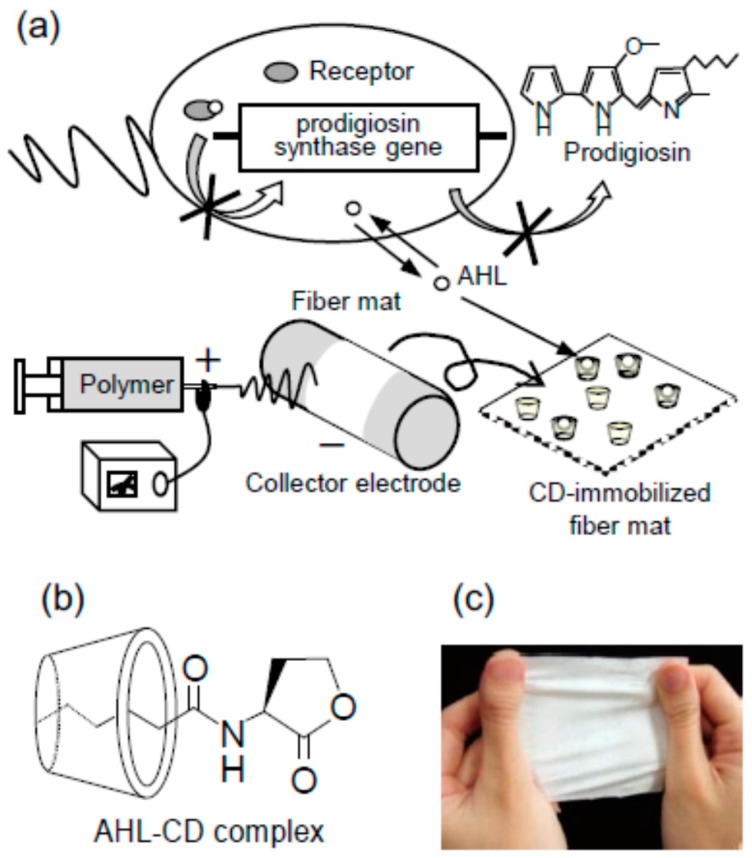
(**a**) Inhibition of Quorum Sensing-dependent prodigiosin production by CD-immobilized electrospun fiber mats; (**b**) Inclusion complex between CD and *N*-acyl-l-homoserine lactone (AHL); (**c**) Image of non-woven fiber mat electrospun from polyvinyl alcohol (PVA) solution. Reproduced from [94] with permission of The Materials Research Society of Japan.

For separation purposes, post-spinning modifications are necessary to guarantee the durability of CD-containing fibers and the maintenance of the filtration efficiency [68]. Owing to the solubility of CDs in aqueous media, leaching of CD molecules takes place during filtration in aqueous media [89]. Cross-linking strategies to obtain water-insoluble CD-based materials have been widely developed to produce networks for environmental applications [90], and these strategies or related ones can be applied to CD-containing electrospun mats to endow them with improved physical and chemical stability. As an example, sericin/βCD/PVA fibers were prepared incorporating also citric acid as crosslinking agent to the blend solution [91]. Incubation of the mats at high temperature triggered the cross-linking process that resulted in water-insoluble nanofibers able to sorb methylene blue from aqueous solutions. Desorption in ethanol and recycling studies showed that the removal efficiency of the mats was maintained after five cycles. Similarly, PVP/βCD fibers cross-linked with glutaraldehyde were shown useful for dye removal from aqueous medium [92]. Importantly, cross-linked nanofibers have been evaluated as imprinted materials for specific recognition of target substances. For example, βCD performed as functional ligand for the capture of naringin that was used as a template. To prepare the imprinted nanofibers the complexes were added to polyvinyl butyral and hexamethylene diisocyanate (used as a cross-linker). The nanofibers that also included an inorganic pore forming agent showed improved binding properties and higher specificity than the non-imprinted nanofibers [93]. In the biomedical field, fibers of PVA and CDs (αCD or HPβCD) internally cross-linked with divinyl sulfone to immobilize the CDs, and then post-crosslinked with glutaraldehyde to make the fibers water insoluble, were proved to be useful for the sorption of *N*-acyl-l-homoserine lactone (AHL) (Figure 4). Thus, the fibers may decrease the concentration of this Quorum Sensing signal altering the communication among bacteria (e.g., *Serratia marcescens*), inhibiting biofilm formation and thus attenuating the virulence [94], as observed for other CD-functionalized systems [95].

Surface functionalization with CDs of preformed fibers is an attractive approach to prepare mats with highly accessible CD cavities on the surface. In blend electrospinning, CD molecules are embedded into the fibers, which can limit their functionality especially for trapping purposes. Surface immobilization allows increasing the amount of CD molecules that could interact with surrounding media [96,97]. Covalent bonding should avoid the leaching process, even when CD-functionalized mats are exposed over an extended period to pollutants-containing water [88]. Mixtures of PLA with amino polyhedral oligomeric silsesquioxanes (POSS-NH_2_) as functional group have been electrospun followed by grafting reaction with βCD via monotosylation [98]. Click chemistry has been applied for the immobilization of azide-βCDs onto propargyl-terminated cellulose acetate fibers, showing enhanced affinity for phenantrene [99]. Although covalent immobilization of CDs provides more efficient functionalization for long term; weaker adsorption of CDs onto the fibers has been also investigated [97,100]. For example, polystyrene fibers containing polydopamine can bind βCD through hydrogen bonding. Such functionalized material showed improved ability to remove contaminants from alkaline solutions [96]. Alternatively, the coating of preformed nanofibers via in situ cross-linking of cyclodextrins has been investigated. Polyester nanofibers were soaked in α-, β- or γCD solutions (10%) also containing citric acid (10%) and sodium hypophosphite (1.2%) for 3 h at 50 °C. Then, the nanofibers were oven-dried at high temperature (105–180 °C) [100]. The water-insoluble coating caused a decrease in surface area and porosity of the mats, but increased the effective capture of phenantrene from water. Nevertheless, there exist also some studies were the advantages of the binding of CDs to the fibers surface are not so clear. For comparative purposes regarding ability to capture formaldehyde, polyacrylonitrile fibers were prepared either with βCD entrapped in the fibers or with a coating of βCD cross-linked with citric acid [101]. Compared to fibers prepared without βCD, incorporation of βCD in either form caused an increase in fiber diameter and a decrease in surface area of mats, which was more remarkable in the case of the coating. Coated fibers adsorbed less formaldehyde and more slowly than βCD-embedded fibers, which has been ascribed to the role that free hydroxyl groups in βCD may play in the interaction with the guest molecules and also to the less rigid conformation of the non-coated fibers [101].

#### 2.1.2. Coaxial processing

Core-shell structures can be prepared applying coaxial electrospinning, which is similar to uniaxial processing except for the nozzle configuration [17,102]. The setup (Figure 1b) involves the use of inner and outer capillary tubes concentrically placed and fed with two different solutions [3,5,25]. Core-shell structures are mainly applied as sustained drug release systems in biomedical field. Active agents hosted into core region are protected by a shell that prevents or reduces the initial burst phenomenon and regulates drug release pattern [3,5]. Drug release depends on the thickness and integrity of core and shell layers. Nevertheless, it should be taken into account that miscibility of solvents used during electrospinning can cause a redistribution of substances from the core to the shell layer, leading to release profiles similar to those recorded for uniaxial electrospun fibers [3,17,44]. Despite possibilities that coaxial technique can open for sustained release, very little work has been done using CDs. As an example, electrospun fibers have been prepared with a core of PCL and a shell of polypseudorotaxanes of four-branches PCL and αCD. The polypseudorotaxanes adopted a nanoplatelet arrangement, which was maintained after electrospinning. The core-shell structure ensured that the hydroxyl groups of αCD were available on the fibers surface for subsequent immobilization of active substances [103].

## 3. Electrospun Mats of Polymer-free Cyclodextrins

Preparation of fibers from compounds of low molecular weight is a challenge due to lack of chain entanglements compared to polymers; nevertheless, several works have reported on the obtaining of fibers based on phospholipids [104] and gemini surfactant [105]. Owing to the intriguing self-assembling features of CDs, strong efforts to prepare fibers using CDs as the only structural component have been made. CDs did not entangle but relevant intermolecular interactions can be established [106,107]. Natural CDs form nano- and micrometric aggregates via intermolecular hydrogen bonding; the aggregates size grows with increasing of CD concentration [108,109]. Readers interested in self-assembling of CDs are referred to excellent reviews elsewhere [109]. In some cases, the presence of guest molecules even promotes the aggregation process. This unique feature of CDs allows for obtaining electrospun fibers from highly concentrated CD-containing solutions [110,111]. Examples of electrospun nanofibers produced from CDs solely or forming inclusion complexes are summarized in Table 1, with indication of solvent used and concentration required for obtaining bead-free nanofibers [110,111,112,113,114,115,116,117,118,119,120].

**Figure 5:**
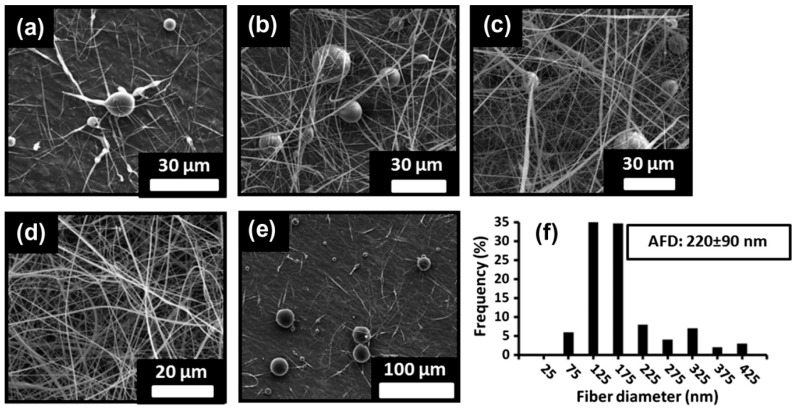
Scanning Electron Microscopy (SEM) images of electrospun fibers prepared with (**a**) 120% (*w/v*), (**b**) 130% (*w/v*), (**c**) 140% (*w/v*), and (**d**) 150% (*w/v*) βCD solutions in 10% NaOH medium, and (**e**) 150% (*w/v*) βCD solutions also containing 20% (*w/w*) urea in 10% NaOH medium. Diameter distribution of fibers prepared with composition (**d**) is shown in (**f**). Reproduced from [110] with permission from Elsevier.

Association among CD molecules occurs through intermolecular hydrogen bonding which limits the interaction with water and causes a decrease in solubility [108,109]. Nevertheless, electrospinning of αCD [110], βCD [110,111] and γCD [112] has been carried out quite similarly to polymeric systems through a careful selection of the solvent nature and the CD concentration that provide solutions of adequate viscosity and conductivity for obtaining bead-free nanofibers. At low CD concentrations, destabilization of electrified jet occurs due to insufficient aggregation, producing bead structures (Figure 5a,b). As CD concentration increases, the aggregates grow in number and size. Thereby, sufficient intermolecular hydrogen bonding and adequate conductivity prevent jet breakage, and bead-free nanofibers can be produced (Figure 5c,d). Rheological properties of native CD solutions shift from Newtonian behavior to viscoelastic solid behavior as the number of aggregates increases [110,111,115]. Interestingly, optimal concentration to obtain bead-free fibers was slightly lower for βCD (150%) than for αCD (160%) when 10% NaOH aq. was used as solvent [110]. The addition of urea caused the de-aggregation of the CDs and a notable decrease in the viscosity of the solution, which in turn led to beaded fibers or just beads (Figure 5e).

Solvent used affects aggregates formation, viscosity, surface tension and conductivity. Therefore, required CD concentration and electrospinning parameters for fiber formation fluctuate hugely in function of the solvent. For example, fibers can be obtained from βCD solution at 60 wt.% concentration in an ionic liquid/*N*,*N*-dimethylformamide (DMF) mixture [111], whereas 150 wt.% βCD was necessary in 10 wt.% NaOH aqueous solution [110]. Increase of DMF ratio in the system decreased viscosity and allowed more elongation of the jet at the same repulsive forces; thus, thinner fibers were obtained. In general, low surface tension solutions are preferable for electrospinning because high surface tension promotes jet instability, but low surface tension does not facilitate fiber formation from low molecular weight solutions [7,18]. The use of 1,1,1,3,3,3-hexafluoro-2-propanol (HFIP) as solvent allowed for producing bead-free fibers of native CDs at much lower concentration; namely, 12.5 wt.% αCD and βCD, and 7.5 wt.% γCD [113]. HFIP improves solubility of CDs and readily evaporates during electrospinning, facilitating the formation of crystalline assemblies of CDs.

Derivatives of native CDs are more soluble but less prone to self-aggregate because of the replacement of hydrogen atoms in hydroxyl groups [108,121]. Nevertheless, electrospun mats containing methyl-β-cyclodextrin (MβCD) [114], HPβCD [34,114,115] and HPγCD [34,114] have been successfully produced using a variety of solvents. In a comparative study, electrospinning of these three CD derivatives was evaluated using water, DMF and dimethylacetamide (DMAc) as solvents [115]. The electrospinning procedure was similar to those implemented for native CDs or polymeric systems, and fiber diameter was influenced by the nature of both the solvent and the CD derivative used. In all cases, aggregates size was larger in DMAc, followed by DMF and water; and hence viscosity required for fiber formation was reached at lower concentration in DMAc, which also provided the thicker fibers. Nevertheless, only DMF allowed for obtaining bead-free fibers with the three derivatives. Polymer-free CD-containing mats usually exhibit fast dissolution rate upon contact with an aqueous medium, and certain applications may require post-spinning modifications in order to that the fibrous structure can be preserved.

**Figure 6:**
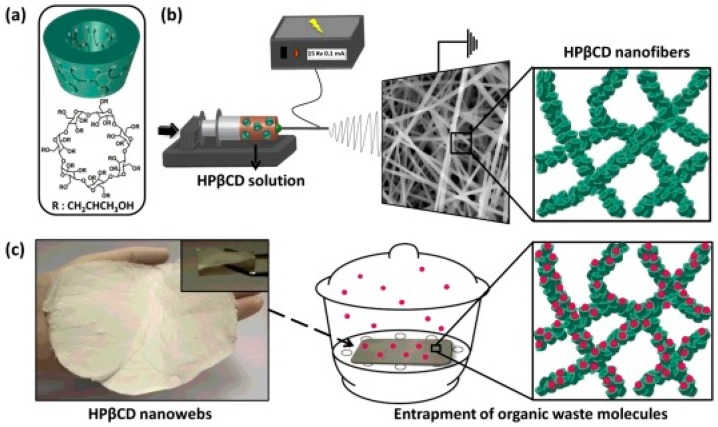
HPβCD solution (**a**) was electrospun to obtain polymer free-fibers (**b**). The obtained mats (**c**) were cut as pieces of 100 mg and exposed to vanillin and benzene vapors inside a desiccator (30 cm diameter × 30 cm height) for 12 h. Reproduced from [34] with permission from Elsevier.

Removal of pollutants from air may be a promising application for fibers prepared with native or derivative CDs solely [34,112]. Combination of large specific surface area of nanofibers (compared to the respective CD powders) and ability to capture volatile organic compounds forming inclusion complexes makes these mats to be very effective for air filtration (Figure 6). Entrapment of aniline and benzene by electrospun HPβCD and HPγCD mats produced from aqueous or DMF solutions has been investigated [34]. HPγCD mats captured lower amount of organic molecules than HPβCD mats because HPγCD has bigger cavity size and less stable interactions can be established with the volatile compounds.

From a practical point of view, electrospinning of inclusion complexes has recently been pointed out as an alternative to freeze-drying for the preparation of amorphous solid complexes that can be rapidly reconstituted in aqueous medium before injection [120]. For this purpose, inclusion complexes of HPβCD with diclofenac sodium were electrospun by means of either uniaxial or blowing-assisted electrospinning. Electroblowing is based on the coaxial nozzle configuration but blowing air stream through the outer capillary tube. Using this later technique, clogging of needle produced by rapid solvent evaporation is avoided and more uniform fibers are obtained [62,120]. The effective drug amorphization during the electrospinning process and the large surface area of the fibers promoted faster dissolution rate of electrospun and electroblown systems. Reconstitution in water of complexes produced by freeze-drying was slower due to recrystallization during the freezing step. Thus, nanofibers can be suitably reconstitutable solids for drug delivery applications.

**Figure 7:**
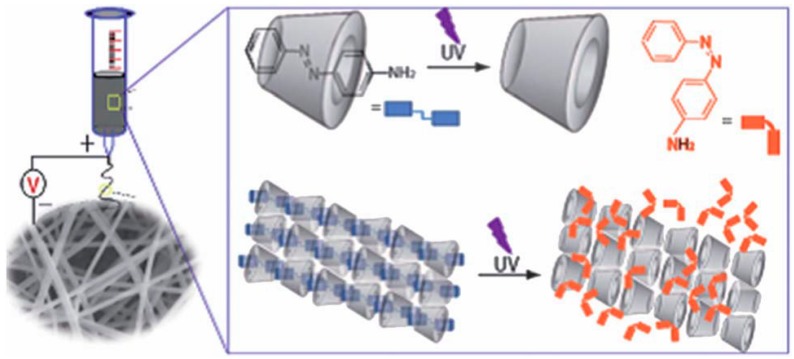
Fibers prepared with HPβCD/4-aminoazobenzene inclusion complexes exhibit UV-responsiveness through conformational changes that the guest molecule undergoes under UV light; the trans isomer transforms into the cis isomer, which is too bulky to remain inside the CD cavity. Reproduced from [119] with permission from The Royal Society of Chemistry.

Electrospinning of polymer-free inclusion complexes allows higher loadings of active agents than those observed for polymeric matrices. However, incorporation of higher amount of drug may disturb the electrospinning process due to changes in solution properties such as conductivity [7]. Electrospun mats from HPβCD, HPγCD and MβCD forming inclusion complexes with vanillin (Table 1) achieved vanillin loadings between 9 and 12% (*w/w*) [118]. Previously, PVA nanofibers including inclusion complexes of native CDs with vanillin attained a maximum content in vanillin of 5% (*w/w* with respect to polymer) [33]. Fiber diameter was thinner for electrospun mats containing inclusion complexes compared to those without active agent due to higher conductivity of solutions [114,118]. Similar results have been reported for geraniol [116], triclosan [117], and 4-aminoazobenzene [119] forming complexes with CD derivatives. Interestingly, the fibers formed from HPβCD and 4-aminoazobenzene showed UV-responsiveness due to that the trans-cis isomerization of the azobenzene group causes a change in the conformation of the guest molecule that determines whether it can be encapsulated in the CD cavity or not (Figure 7) [119]. 4-Aminoazobenzene forms inclusion complexes in the trans state (relaxed conformation) whereas the cis isomer (formed after UV irradiation) is too bulky. When the UV radiation was applied during electrospinning of HPβCD/4-aminoazobenzene 1:0.7 mol/mol complexes, the decomplexation process caused an increase in the diameter of the fibers. When the UV radiation was applied after mats formation, the fibers notably modified their topography [119]. More complex photo-responsive behaviour was observed for fibers prepared with βCD coupled with an spiropyran derivative and optionally containing poly(methacrylic acid) to modulate the rate of isomerization [122]. The fibers showed reversible photo-responsive behavior under cycles of visible light-UV radiation.

## 4. Electrospun Mats of Cyclodextrin-based Polymers 

Poly(cyclodextrins) prepared through cross-linking of native or derivative CDs or polymerization of CD monomers have been also explored as components of electrospun nanofibers. Both uniaxial and coaxial fibers were prepared from a cyclodextrin-epichlorhydrin polymer (polyCD) and poly(methacrylic acid) (PMAA) to investigate the ability to sustain the release of propranolol [123]. The fibers were cured at 170 °C for 48 h to trigger crosslinking formation between the two polymers, which caused the mats to become insoluble in water. Uniaxial fibers prepared with PMAA/polyCD (80:20 *w/w*) and (60:40 *w/w*) released 30% and 35% loaded drug in 8 h, while PMAA solely fibers released 100% dose in 15 min. Coaxial fibers having a core of polyCD-propranolol complexes and a shell of PMAA showed even more sustained release, although the entire dose may be not released [123]. In a different approach, uniaxial fibers of polyCD-fluconazole inclusion complexes mixed with PCL or PVP were provided with a hydrophobic coating of poly(hexamethyldisiloxane) (polyHMDSO) for modulating fibers dissolution and drug release rate [124]. The coating was applied under mild conditions plasma polymerization. Differently to non-coated mats that rapidly released the whole amount of drug, polyHMDSO-coated fibers extended drug release for 24 hours. HMDSO-coated fluconazole-loaded fibers efficiently inhibited in vitro the growth of *Candida albicans* [124].

Anionic HPβCD-citric acid polymer has been mixed with chitosan to form polyelectrolyte complexes that can render insoluble nanofibers with antibacterial activity [125]. Triclosan first formed inclusion complexes with the polyHPβCD and then the complexes in solution were mixed with chitosan. As controls chitosan fibers containing triclosan or common HPβCD:triclosan complexes were prepared. The obtained fibers were cured at 90°C for 4 h to make them less water-soluble. Fibers prepared with the chitosan-polyHPβCD polyelectrolyte complexes swelled less both at pH 5.5 and 7.4 and released the drug more slowly than the controls. Importantly, the polyelectrolyte complexes fibers showed superior antimicrobial activity against *S. aureus* and *E. coli* for prolonged periods of time [125].

## 5. Conclusions and Future Outlook

Electrospun mats can be endowed with a variety of useful functions by means of the incorporation of CDs. These small oligosaccharides can fully develop their ability to form inclusion complexes either embedded into the nanofiber matrix or once grafted onto the fibers surface. In most cases, incorporation of CDs aims to modulate drug release by forming inclusion complexes with a target drug, usually accelerating the dissolution process in water or delaying the release of volatile compounds to the air. Interestingly, novel applications are being envisioned for polymer nanofibers containing free CDs in which the CDs can play a variety of roles, ranging from simple cross-linking agents in mats including stimuli-responsive polymers, to ligands for specific recognition for affinity separations, or to building blocks that can facilitate the construction of mat-by-mat assemblies that may result in ad hoc scaffolds or self-healing materials [126]. Two main advances in the field can be pointed out. The first one refers to the feasibility of using CDs (or their inclusion complexes) as the only component of electrospun fibers, which is opening novel ways of formulation of fast dissolving amorphous solids for several administration routes. A better knowledge about the self-aggregation mechanisms of CDs into the common solvents used for electrospinning as well as the repercussions of such aggregation on critical properties such as viscoelasticity and conductivity should pave the way for a formulator-friendly design of the electrospun conditions (avoiding toxic solvents and harsh pH or temperature conditions). The second advance refers to the possibilities that CD polymers may offer if a good combination of chain entanglement and complex formation is attained. Differently to the case of polypseudorotaxanes that only allow using the hydroxyl groups of CDs to immobilize target substances, CD polymers can make use also of the cavities acting as multipurpose tools. In this regards, design of CD polymers that can be electrospinnable by themselves should be still improved. Overall, electrospun nanofibers appear as an excellent format for the fully exploitation of the amazing multifaceted tasks that CDs can develop.

## Figures and Tables

**Table 1:** Composition of bead-free electrospun mats obtained from CDs or CD-inclusion complexes (IC) and their applications.

CD or IC	Concentration (% *w/v*)	Solvents (*v/v*)	Application	Reference
αCD	160	10% NaOH		[110]
βCD	150			
βCD	60	DMF/1-ethyl-3-methylimidazolium acetate (3:7 or 4:6)		[111]
γCD	140	DMSO/Water (50:50)	Entrapment volatile organic compounds (aniline, toluene)	[112]
αCD	12.5	1,1,1,3,3,3-Hexafluoro-2-propanol (HFIP)		[113]
βCD	12.5			
γCD	7.5			
MβCD	140; 160	Water		[114]
	140; 160	DMF		
	160	DMAc		
HPβCD	160	Water		
	120	DMF		
	120	DMAc		
HPγCD	160	Water		
	125	DMF		
	125	DMAc		
HPβCD	160	Water	Entrapment of volatile organic compounds (aniline, benzene)	[34]
	120	DMF		
HPγCD	160	Water		
	125	DMF		
HPβCD	61.4	DMF		[115]
(HPβCD; HPγCD; MβCD)-Geraniol-IC	200	Water	Prolonged releasing systems with antioxidant and antibacterial activity	[116]
(HPβCD; HPγCD)-Triclosan-IC	160	Water	Wound dressings with antibacterial activity	[117]
HPβCD-Vanillin-IC	160	Water	Food or pharmaceutical products	[118]
	120	DMF, DMAc		
HPγCD-Vanillin-IC	160	Water		
	125	DMF, DMAc		
MβCD-Vanillin-IC	160	Water		
	160	DMF, DMAc		
HPβCD-4-aminoazobenzene-IC	130	Water	Drug delivery, sensors	[119]
HPβCD-diclofenac	45	Ethanol	Fast dissolving solid complexes	[120]

DMAc, dimethylacetamide; DMF, dimethylformamide; DMSO, dimethylsulfoxide; HPβCD, hydroxypropyl-β-cyclodextrin; HPγCD, hydroxypropyl-γ-cyclodextrin; MβCD, methyl-β-cyclodextrin.

## Abbreviations

The following abbreviations are used in this manuscript:

AHL: *N*-acyl-L-homoserine lactone
CD: cyclodextrin
CFM: chloroform
CNTs: multiwalled carbon nanotubes
DMAc: dimethylacetamide
DMF: dimethylformamide
DMSO: dimethylsulfoxide
ECM: extracellular matrix
HCPT: hydroxycamptothecin
HMDSO: hexamethyldisiloxane
HFIP: 1,1,1,3,3,3-hexafluoro-2-propanol
HPC: hydroxypropyl cellulose
HPβCD: hydroxypropyl-β-cyclodextrin
HPγCD: hydroxypropyl-γ-cyclodextrin
MβCD: methyl-β-cyclodextrin
PAA: poly(acrylic acid)
PBS: phosphate buffer saline
PCL: poly(ε-caprolactone)
PDADMAC: poly(diallyldimethylammonium chloride)
PEG: polyethylene glycol
PELA: poly(DL-lactic acid)-poly(ethylene glycol)
PEO: poly(ethylene oxide)
PLA: polylactic acid
PMAA: poly(methacrylic acid)
PMMA: poly(methyl methacrylate)
POSS-NH_2_: amino polyhedral oligomeric silsesquioxanes
PVA: polyvinyl alcohol
PVdF: polyvinylidene fluoride
PVP: polyvinyl pyrrolidone
SBEβCD: sulfobutylether-β-cyclodextrin
